# Comparisons of Mechanical Power and Respiratory Mechanics in Pressure-Controlled Ventilation and Volume-Controlled Ventilation during Laparoscopic Cholecystectomy in Elderly Patients

**DOI:** 10.3390/jpm13020201

**Published:** 2023-01-23

**Authors:** Youn Yi Jo, Young Jin Chang, Dongchul Lee, Yong Beom Kim, Junsu Jung, Hyun Jeong Kwak

**Affiliations:** Department of Anesthesiology and Pain Medicine, Gachon University College of Medicine, Gil Hospital, 21, Namdong-daero 774 Beon-Gil, Namdong-gu, Incheon 21565, Republic of Korea

**Keywords:** elderly, laparoscopy, pressure-controlled ventilation, volume-controlled ventilation, mechanical power

## Abstract

We compared the effects of pressure-controlled volume-guaranteed ventilation (PCV) and volume-controlled ventilation (VCV) on respiratory mechanics and mechanical power (MP) in elderly patients undergoing laparoscopy. Fifty patients aged 65–80 years scheduled for laparoscopic cholecystectomy were randomly assigned to either the VCV group (*n* = 25) or the PCV group (*n* = 25). The ventilator had the same settings in both modes. The change in MP over time was insignificant between the groups (*p* = 0.911). MP significantly increased during pneumoperitoneum in both groups compared with anesthesia induction (IND). The increase in MP from IND to 30 min after pneumoperitoneum (PP30) was not different between the VCV and PCV groups. The change in driving pressure (DP) over time were significantly different between the groups during surgery, and the increase in DP from IND to PP30 was significantly higher in the VCV group than in the PCV group (both *p* = 0.001). Changes in MP during PCV and VCV were similar in elderly patients, and MP increased significantly during pneumoperitoneum in both groups. However, MP did not reach clinical significance (≥12 J/min). In contrast, the PCV group had a significantly lower increase in DP after pneumoperitoneum than the VCV group.

## 1. Introduction

There are many situations in which a mechanical ventilator must be applied for life support. However, mechanical ventilation itself causes ventilator-induced lung injury (VILI) [1], which is determined by the amount of energy delivered to the lungs. Mechanical power (MP) can substitute this energy transferred to the lungs [1,2] and is affected by several factors, including tidal volume (Vt), pressure delivered to the alveoli, and respiratory rate (RR) [2]. An increase in MP is associated with high morbidity and mortality in critically ill patients receiving mechanical ventilation [3]. In a previous analysis of 8207 patients, MP was an independent prognostic factor for in-hospital mortality, 30-day mortality, and length of hospital stay [3]. Even at low Vt, high MP increased mortality and morbidity, and was positively correlated with increased mortality when MP was higher than 17 J/min [3].

Intraoperative mechanical ventilation for general anesthesia has shown comparable results to those of critically ill patients [4]. In a previous clinical study of patients who underwent major non-cardiothoracic and non-intracranial surgeries, a higher MP was associated with a higher risk of postoperative pulmonary complications, including acute respiratory failure [4]. Another retrospective analysis of 230,767 non-cardiac surgical cases also showed similar results [5]. In their study, intraoperative value of MP (median [interquatile range, IQR]) was significantly higher in patients who required reintubation due to postoperative respiratory failure within 7 days after surgery than in patient who did not (7.67 [5.64–10.11] J/min vs. 6.62 [4.62–9.10] J/min) [5]. In addition, when performed adjusted analysis for known factors inducing VILI such as Vt, driving pressure (DP), and RR, MP remained an independent risk factor for postoperative respiratory failure [5]. 

The most basic modes of mechanical ventilation are volume-controlled ventilation (VCV) and pressure-controlled ventilation (PCV). PCV has the advantages of lower peak airway pressure and improved respiratory compliance compared to VCV in laparoscopic surgery. However, PCV has the weakness of continuously monitoring and changing the pressure setting in order to keep Vt constant in a situation where airway pressure can change rapidly, such as in the pneumoperitoneum [6]. Recently, the PCV-volume guarantee (PCV-VG) mode has been preferred as a mode that compensates for the disadvantages of PCV, in which Vt is not guaranteed. PCV-VG provides lower peak inspiratory pressure (PIP) with improved dynamic lung compliance (Cdyn) than VCV, even in laparoscopic surgery [7]. A recent meta-analysis of 17 publications involving a total of 929 participants demonstrated that PCV-VG provided beneficial effects on respiratory mechanics with no relative disadvantages over VCV in both one-lung and two-lung ventilated patients undergoing non-cardiac surgery [8]. In elderly patients (≥65 years) who underwent thoracotomy, PCV-VG had significantly lower PIP and improved Cdyn during one-lung ventilation than VCV [6]. In addition, the inflammatory responses to mechanical ventilation were significantly lower in the PCV-VG mode than in the VCV mode [9]. 

We hypothesized that PCV-VG might be associated with lower MP than VCV in elderly patients during laparoscopic surgery. Thus, we compared the effects of two ventilation modes (PCV-VG vs. VCV) on MP and respiratory mechanics in elderly patients undergoing laparoscopic cholecystectomy.

## 2. Materials and Methods

Prior to conducting this prospective study, permission was obtained from the Institutional Review Board of Gachon University Gil Hospital (GFIRB2021-117), and the study was registered with the Clinical Research Information Service of the Korean government (ref. cris.nih.go.kr accessed on 3 May 2021; KCT 0006144). All participants provided written informed consent prior to surgery. This study involved 50 elderly patients aged 65–80 years who were scheduled for elective laparoscopic cholecystectomy and had an American Society of Anesthesiologists (ASA) physical status of 2 (between May 2021 and March 2022). This study excluded patients with a history of active pulmonary disease, uncontrolled cardiovascular morbidity, moderate-to-severe restrictive or obstructive pulmonary disease, and high body mass index (BMI ≥ 30 kg/m^2^). Patients with subcutaneous emphysema during pneumoperitoneum were excluded from the analysis. Patients were randomly assigned according to whether VCV mode (VCV group, *n* = 25) or PCV-VG mode (PCV group, *n* = 25) was applied using Excel 2013 (Microsoft Office, Redmond, WA, USA) without stratification. When the surgery schedule was uploaded, the inclusion and exclusion criteria were checked and the anesthesiologist trainee who did not conducting the study and outcome assess, decided the enrolled participants and informed consent was obtained. Participants, care providers, and outcome assessors were not aware of the assigned group except for the anesthesiologist conducting the study. The anesthesiologist conducting the study received a sealed envelope containing the group assignment according to the list created by the coordinator who did not participate in the research and conducted the research.

Premedication was not administered for all patients. In the operating room, standard anesthesia monitoring such as bispectral index (BIS), pulse oximetry, electrocardiography, and non-invasive blood pressure monitoring were employed. Lidocaine of 1 mg/kg, propofol of 1–1.5 mg/kg, alfentanil of 10–15 µg/kg, and rocuronium of 0.8 mg/kg were used for anesthetic induction, while sevoflurane of 0.8–1.2 vol% and remifentanil of 0.1–0.2 µg/kg/min were used to maintenance a BIS score within a range of 40–60. Each group chose the VCV mode (VCV group) or the PCV-VG mode (PCV group) according to their assignment. The ventilator (Datex Ohmeda S5 Avance; GE Healthcare, Madison, WI, USA) had the same settings in both modes: a Vt of 6–7 mL/kg of ideal body weight (0.919 × (height in cm − 152.4) + 50] for men, or 0.919 × (height in cm − 152.4) + 45.5 for women), a positive end-expiratory pressure (PEEP) of 5 cmH_2_O, an inspiratory to expiratory (I/E) ratio of 1:1, inspiratory pause of 10% of inspiratory time in the VCV group only, and an inspired oxygen fraction (FiO_2_) of 0.5, with oxygen and medical air. The RR was adjusted to maintain a target end-tidal carbon dioxide tension (E_T_CO_2_) of 35–40 mmHg. Measured respiratory parameters including Vt, RR, PIP, plateau pressure (Pplat), mean airway pressure (Pmean), PEEP, E_T_CO_2_, and SpO_2_, and hemodynamic variables including blood pressure and heart rate were recorded at 10 min after anesthesia induction in the supine position (IND), 15 and 30 min after pneumoperitoneum induction (PP15 and PP30, respectively) in the reverse Trendelenburg position, and at the end of the operation in the supine position (END). 

MP values were calculated as follows: 0.098∙RR∙Vt∙[PIP − 0.5 (Pplat-PEEP)] in the VCV group and 0.098∙RR∙Vt∙[PEEP + pressure above PEEP] in the PCV group. In these equations, 0.098 is the conversion factor for converting from units of cmH_2_O∙L/min to J/min, unit of Vt is liter, and units of airway pressures and PEEP is cmH_2_O [10]. DP values were calculated as follows: Pplat-PEEP in the VCV group and PIP-PEEP in the PCV group [11]. Dynamic DP and dynamic MP values were calculated as follows: PIP-PEEP and 0.098∙RR∙Vt∙[PIP − (0.5 × dynamic DP)] for both groups [12]. Cdyn was calculated as Vt/(PIP-PEEP) in both groups. Intra-abdominal pressure was limited at 12–15 mmHg during pneumoperitoneum. The position was changed from supine to a 30° reverse Trendelenburg position immediate after intra-abdominal CO_2_ insufflation. Patients was monitored for respiratory complications including desaturation events during the hospital stay after surgery.

The primary outcome variable was MP after CO_2_ pneumoperitoneum for laparoscopy. The sample size was calculated based on a preliminary study, which showed that the MP (mean ± standard deviation, SD) after pneumoperitoneum was 6.9 ± 1.4 J/min during VCV 30 min after pneumoperitoneum in the reverse Trendelenburg position in 10 patients who underwent elective laparoscopic cholecystectomy (≥65 years). To demonstrate a difference of 20% in MP during PCV, with a power of 90% and an α-error of 0.05, 21 participants were required for each group. We included 25 patients per group to account for a dropout rate of 20%.

Statistical analyses were performed using SPSS version 22.0 (SPSS Inc., Chicago, IL, USA). Values are presented as mean ± SD, number of patients, or median (interquartile range). The normality of the distribution of continuous variables was assessed using the Kolmogorov–Smirnov test. Intergroup differences in continuous variables were assessed using an independent *t*-test or Mann–Whitney U test. Intergroup differences in categorical data were assessed using the chi-square test or Fisher’s exact test, as appropriate. Changes in respiratory variables over time were analyzed using repeated-measures ANOVA variance. Statistical significance was set at *p* < 0.05.

## 3. Results

**Figure 1:**
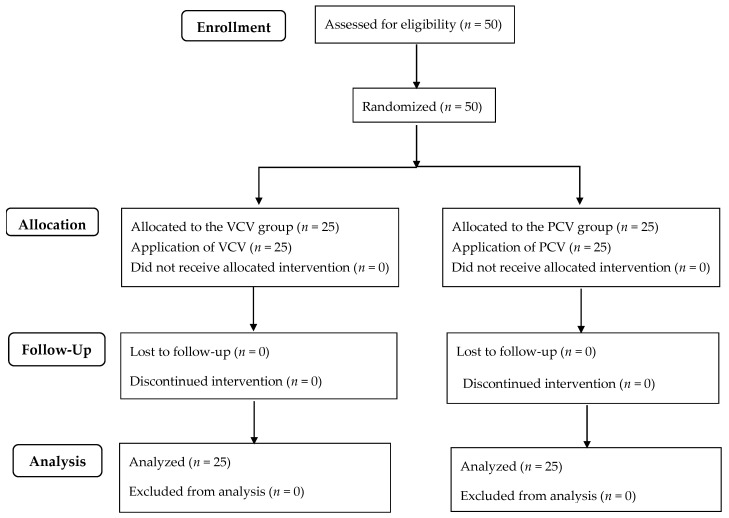
Flow diagram of patient’s allocation. VCV, volume-controlled ventilation; PCV, pressure-controlled ventilation-volume guarantee.

Fifty patients were enrolled, and none was excluded from the analysis (Figure 1). Patient characteristics were similar between the VCV and PCV groups (Table 1). The mean age of the participants was 71.0 ± 4.5 years. Intraoperative data including anesthesia, operation, and pneumoperitoneum times were similar between the VCV and PCV groups (*p* = 0.086, 0.369, and 0.247, respectively). Postoperative hospital stay was similar between VCV and PCV groups (*p* = 0.822).

**Figure 2:**
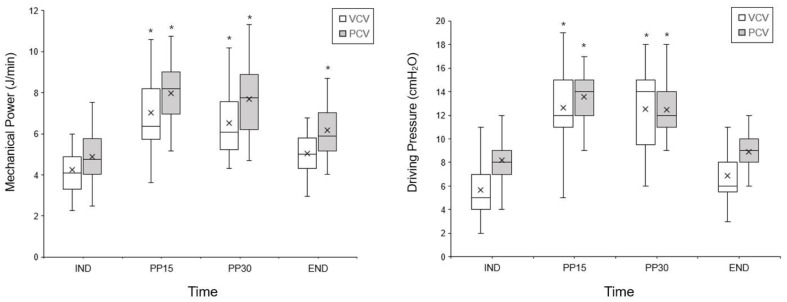
The change in mechanical power (MP) and driving pressure (DP) during the surgery. VCV, volume-controlled ventilation; PCV, pressure-controlled ventilation-volume guarantee; IND, 10 min after anesthesia induction; PP15 and PP30, 15 and 30 min after pneumoperitoneum induction; END, the end of surgery. There was no significant difference in the change over time in the MP (*p* = 0.911), whereas the change over time in the DP was significantly different between the groups (*p* = 0.001). Error bars, boxes, middle lines, and x represent the range (minimum-maximum), interquartile range, median, and mean, respectively. * *p* < 0.05, vs. IND within the group.

Figure 2 illustrates the changes in MP and DP during surgery. The change in MP over time was not significantly different between the groups (*p* = 0.911). Compared to IND, MP significantly increased at PP15 and PP30 in the VCV group and at PP15, PP30, and END in the PCV group. There was no difference in the increment of MP from IND to PP30 between the VCV and PCV groups (2.5 ± 1.7 J/min vs. 2.7 ± 1.9 J/min, *p* = 0.721). Moreover, there was no difference in the relative increment of MP from IND to PP30 ((PP30-IND)/IND) between the VCV and PCV groups (0.71 ± 0.48 vs. 0.66 ± 0.45, *p* = 0.707).

No patient showed a high MP of over 12 J/min throughout the surgery in either group. The change in DP over time was significantly different between the groups (*p* = 0.001). Compared to IND, DP significantly increased at PP15 and PP30 in both groups. The increment of DP from IND to PP30 was significantly higher in the VCV than in the PCV group (6.9 ± 2.7 cmH_2_O vs. 4.3 ± 2.5 cmH_2_O, *p* = 0.001). The relative increment of DP from IND to PP30 ((PP30-IND)/IND) was significantly higher in the VCV group than in the PCV group (0.75 ± 0.41 vs. 0.47 ± 0.23, *p* = 0.005).

No cases of postoperative desaturation occurred during the study period.

The dynamic MP and dynamic DP are shown in Table 2. The changes in dynamic MP and dynamic DP over time were significantly different between the groups (*p* = 0.007, and 0.001, respectively). Compared to IND, dynamic MP was significantly increased at PP15 and PP30 in the VCV group (all *p* < 0.001), and increased at PP15, PP30, and END in the PCV group (*p* < 0.001, <0.001, and 0.046, respectively). Compared to IND, dynamic DP was significantly increased at PP15 and PP30 in both groups (all *p* < 0.001). The relative increment of dynamic MP from IND to PP30 ((PP30-IND)/IND) was higher in the VCV than in the PCV group without statistical significance (0.75 ± 0.48 vs. 0.52 ± 0.39, *p* = 0.062). The relative increment of dynamic DP from IND to PP30 ((PP30-IND)/IND) was higher in the VCV group than in the PCV group without statistical significance (0.84 ± 0.26 vs. 0.71 ± 0.32, *p* = 0.106).

The intraoperatively measured respiratory parameters are shown in Table 3. Except for PIP (*p* = 0.001), the changes in Vt, RR, minute volume, Pmean, E_T_CO_2,_ and Cdyn over time did not differ significantly between the groups (*p* = 0.089, 0.156, 0.265, 0.762, 0.552, and 0.524, respectively). Compared to IND, PIP significantly increased at PP15 and PP30 in both groups (all *p* < 0.001). The increment of PIP from IND to PP30 was significantly higher in the VCV than in the PCV group (6.7 ± 2.8 cmH_2_O vs. 4.2 ± 2.5 cmH_2_O, *p* = 0.002). Compared to IND, Pmean significantly increased, whereas Cdyn decreased at PP15 and PP30 in both groups (all *p* < 0.001). Compared with IND, RR significantly increased at PP15, PP30, and END only in the VCV group (*p* = 0.002, 0.001, and 0.004, respectively). Compared to IND, E_T_CO_2_ significantly increased at PP15, PP30, and END in both groups (all *p* < 0.001).

There were no significant differences in the changes over time in the mean arterial pressure and heart rate between the groups (*p* = 0.598 and 0.266, respectively). When compared to IND, mean arterial pressure increased significantly at PP15 in both groups (all *p* < 0.05), whereas heart rate did not change significantly during surgery in either group (data not shown).

## 4. Discussion

In this study, changes in MP during the VCV and PCV-VG modes were similar in elderly patients undergoing laparoscopic cholecystectomy, and MP increased significantly after pneumoperitoneum in both groups. By contrast, in both groups, these increases did not reach clinically significant levels (≥12 J/min) and did not lead to clinically significant postoperative desaturation. However, the PCV-VG mode had significantly lower increases in DP and PIP after pneumoperitoneum than the VCV mode. 

Mechanical forces such as volume, pressure, and flow created by the interaction between the respiratory system and the ventilator can further damage the lung, which is called VILI [13]. Several strategies have been proposed to reduce VILI, based primarily on Vt [14], DP [15], and PEEP [16]. In addition, RR [17] and inspiratory flow [18] have also been suggested as factors that can promote VILI. To estimate the contribution of the various ventilator-related causes of lung injury, MP has been proposed by combining all these factors in a single physical variable [19]. MP is the energy delivered to the respiratory system over time and is the product of the absolute proximal airway pressure and the related changes in Vt and RR [19]. To facilitate the calculation of MP, a simplified form instead of the original equation was proposed, requiring only measurements of Pplat, Vt and PEEP [20]. However, this equation can be used only during VCV mode with constant inspiratory flow in sedated patients with no spontaneous respiratory drive. During PCV, in which the airway pressure is held constant, and the flow is decelerated during the mechanical breath, two alternative equations have been suggested [21,22]. The previous study has reported that measuring MP with the geometric method that is considered the reference standard and comparing them with the surrogate formula, the surrogate formula for both the VCV and PCV modes showed sufficient accuracy for their use in clinical practice [10]. In this study, we used the surrogate formula validated in a previous study by Chiumello et al. [10] for calculation of MP in the PCV group. 

The MP calculation formulas are functions consisting of respiratory parameters such as Vt, RR, DP, PEEP, I/E ratio, and airway flow, and the effect of each respiratory parameter on MP might be weighted differently [19]. In an earlier animal study, different MP values were applied to the respiratory system by varying the RR while keeping the Vt and transpulmonary pressure constant to identify an MP threshold for lung injury in healthy pigs [2]. They reported that lung injury correlated with changes in MP and that pulmonary edema only occurred in transpulmonary MP delivered at 12.1 J/min or higher [2]. In addition, MP was divided into low and high groups based on 12 J/min, and the increase in lung elastance and the decrease in PaO_2_/FiO_2_ ratio were significantly higher in the high MP group than in the low MP group at the end of the study than at the start of the study [2]. These results show that high MP might have a negative effect on lung physiology. In this study, there was no high MP > 12 J/min throughout the surgery and no significant oxygen desaturation or pulmonary morbidity after surgery in either group; thus, both the VCV and PCV-VG modes could be considered suitable for laparoscopic surgery in elderly patients. 

Recently, Pplat and DP are easily used clinical variables to monitor whether a certain setup of mechanical ventilation is protective or not [15]. A major advantage of DP is that it is extremely simple to calculate and highly correlated with mortality. In this study, the change in DP was significantly different between VCV and PCV groups, although both groups showed similar changes in MP. There could be two reasons for the difference in DP changes. First, because Pplat could not be measured in the PCV group in this study, the calculation formula in the PCV group was modified with the difference between PIP and PEEP and not the difference between Pplat and PEEP. Second, PCV has the advantage of lowering PIP compared to VCV when the same Vt is delivered [23]. In a recent meta-analysis, PCV significantly lowered PIP with higher respiratory compliance during laparoscopy [24]. The high initial flow rate of PCV permits rapid and homogeneous alveolar recruitment and delivers Vt more quickly at the beginning of the inspiratory phase in PCV than in VCV [25]. In this study, the PCV group showed lower PIP with relatively better Cdyn, despite a similar Vt than the VCV group. In particular, considering the fact that pneumoperitoneum can induce a significant increase in intra-abdominal pressure, which may lead to increments in airway pressure and deterioration of respiratory compliance, PCV-VG is considered a better choice than VCV for laparoscopy in elderly patients.

Recent studies have reported that PCV has more beneficial effects on the respiratory system than VCV in elderly patients [26,27,28], although there has been controversy over whether PCV or VCV is better for mechanical ventilator care during general anesthesia. A previous study has shown that during one-lung ventilation in elderly patients, PCV could provide significantly improved oxygenation and lower PIP than VCV, as well as a higher postoperative PaO_2_/FiO_2_ ratio and shorter intensive care unit and hospital stays [26]. In patients who underwent laparoscopic colectomy (mean age > 60 years), Cdyn and static compliance were significantly higher, and PIP was lower in the PCV group than in the VCV group during pneumoperitoneum [27]. In addition, in their study [27], the overall plasma soluble receptor for advanced glycation end-products (sRAGE) and S100 calcium-binding protein A12 (S100A12), which are biomarkers that increase in acute lung injury, were significantly higher in the VCV group than in the PCV group [27]. Lower PIP and higher Cdyn are evaluated positively compared to VCV in elderly patients undergoing laparoscopic surgery [28]. Our results of lower PIP and higher Cdyn in the PCV group than in the VCV group are consistent with those of previous studies [26,27,28]. 

In the classical lung-protective ventilation strategy, low Vt and high PEEP have been accepted as standards, but in recent studies, high DP rather than the level of PEEP has been reported as a factor related to postoperative pulmonary complications [29]. A recent meta-analysis demonstrated that increases in DP by 1 unit was associated with the development of postoperative pulmonary complications [29]. During constant Vt, increased DP, which is induced by increasing PEEP, might lead to increased alveolar strain and aggravate negative effects on the respiratory system [30]. In this study, DP increased significantly after pneumoperitoneum in both groups, and the increment in DP from IND to PP30 was significantly higher in the VCV group than in the PCV group. The static lung compliance (Cstat) and DP were inversely proportional. 

Optimization of respiratory mechanics in elderly patients is very important because physiological and biochemical changes caused by aging can lead to deteriorate respiratory function and increase postoperative pulmonary complications [31]. Furthermore, carbon dioxide insufflation for laparoscopic surgery is accompanied by not only hemodynamic changes but also airway pressure elevation, hypercarbia, and decreased pulmonary compliance. Therefore, strategies that can improve respiratory mechanics in laparoscopic surgery in elderly patients needed to be considered [32]. In this study, both groups received the ventilation with an I/E ratio 1:1 instead of the ventilation with a conventional ratio 1:2. There were previous studies that increasing the inspiratory time by setting the I:E ratio to 1:1 or 2:1 is better for gas exchange [33]. Therefore, we chose an I:E ratio of 1:1 for lung protective ventilation in elderly patients. 

This study had some limitations. First, because the VCV and PCV-VG modes have different flow curve characteristics, it is impossible to apply the same formula for the two ventilatory modes, and a simple arithmetic comparison may be meaningless in this study. The MP includes both elastic and resistive components. In this study, in the PCV group, PIP was equivalent to Pplat, while in the VCV group, PIP was higher than Pplat owing to the resistive component. As it is unclear whether the elastic and resistive components of power have the same biological impact, further studies on the relationship between ventilation modes, MP, and lung injury may be warranted. Second, we cannot measure Pplat in the PCV group because inspiratory hold is not available in our anesthetic machine during PCV-VG mode. It would be most ideal if Pplat was measured using an inspiratory hold in both groups, because PIP and Pplat in the PCV group are not exactly the same, and PIP (pressure above PEEP) does not reflect alveolar pressure at the end of inspiration in contrast to Pplat. Further study measuring Pplat properly for DP and MP calculations in both groups may be needed to make firm conclusions. However, in this study, there was little concern about excessive resistive load because the prolonged inspiratory time was set (I:E ratio was set at 1:1), patients with pulmonary disease were excluded, and neuromuscular blocking agent was used. Therefore, in this study, as the flow converges to zero at the end of the inspiratory phase, Pplat can almost coincide PIP at the end of inspiration [34]. We also calculated dynamic MP and dynamic DP to compensate for not being able to directly measure Pplat in the PCV group. Third, although we studied only elderly patients, because we excluded severe underlying diseases, the results cannot be extended to all elderly patients. Fourth, posture during laparoscopic surgery has markedly different effects on respiratory physiology [35]. Since we conducted a study on laparoscopic cholecystectomy, which is performed in the reverse Trendelenburg position, it is difficult to apply our results to patients whose lung physiology has deteriorated due to the Trendelenburg position. Moreover, since laparoscopic cholecystectomy is a relatively short and simple laparoscopic surgery, additional research may be needed in major laparoscopic surgery, which requires a long operation time. 

In conclusion, the changes in MP during PCV-VG and VCV were similar in elderly patients undergoing laparoscopic cholecystectomy, and MP increased significantly during pneumoperitoneum in both groups. In contrast, the PCV group had significantly lower increases in DP and PIP after pneumoperitoneum than did the VCV group.

## Figures and Tables

**Table 1:** Patient characteristics and perioperative data.

	VCV (*n* = 25)	PCV (*n* = 25)	*p* Value
Age, years	70.3 ± 4.7	71.8 ± 4.2	0.258
Gender, M/F	10/15	17/8	0.384
Weight, kg	62.2 ± 9.9	63.5 ± 9.3	0.638
Height, cm	160.9 ± 7.3	161. ± 8.0	0.941
ASA PS, I/II	4/21	6/19	0.363
Anesthesia time, min	92.5 ± 23.1	81.8 ± 19.9	0.086
Operation time, min	63.0 ± 22.9	57.4 ± 21.0	0.369
Pneumoperitoneum time, min	48.6 ± 21.5	42.0 ± 18.1	0.247
Postoperative hospital stay, days	3 (3–4)	3 (3–4)	0.822

Values are presented as mean ± SD, number of patients, or median (interquartile range). VCV, volume-controlled ventilation; PCV, pressure-controlled ventilation-volume guarantee; ASA PS, American Society of Anesthesiologists Physical Status.

**Table 2:** The change in dynamic mechanical power and dynamic driving pressure during the surgery.

Variables	Group	IND	PP15	PP30	END	Group * Time
Dynamic mechanical power, J/min	VCV	3.6 ± 0.9	6.2 ± 1.7 *	5.9 ± 1.6 *	4.5 ± 1.1	0.007
	PCV	3.5 ± 0.8	5.1 ± 1.0 *	4.9 ± 1.1 *	4.2 ± 0.8 *	
Dynamic driving pressure, cmH_2_O	VCV	9.2 ± 2.5	16.6 ± 2.8 *	16.0 ± 2.7 *	10.1 ± 1.9	0.001
	PCV	8.2 ± 2.2	13.6 ± 2.7 *	12.5 ± 2.5 *	8.9 ± 1.6	

Values are presented as the mean ± SD. VCV, volume-controlled ventilation; PCV, pressure-controlled ventilation-volume guarantee; IND, 10 min after anesthesia induction; PP15 and PP30, 15 and 30 min after pneumoperitoneum induction; END, the end of surgery. Group * time, aspect of parameter change over time; * *p* < 0.05, vs. IND within the group.

**Table 3:** Intraoperative measured respiratory parameters during the surgery.

Variables	Group	IND	PP15	PP30	END	Group * Time
Tidal volume, mL/min	VCV	347 ± 52	352 ± 48	347 ± 47	345 ± 54	0.089
	PCV	354 ± 67	370 ± 56	371 ± 55	372 ± 59	
Respiratory rate, breaths/min	VCV	11 ± 2	13 ± 2 *	13 ± 2 *	13 ± 2 *	0.156
	PCV	11 ± 1	12 ± 2	12 ± 2	12 ± 2	
Peak inspiratory pressure, cmH_2_O	VCV	14 ± 2	22 ± 3 *	21 ± 3 *	15 ± 2	0.001
	PCV	13 ± 2	19 ± 3 *	17 ± 3 *	14 ± 2	
Minute volume, L/min	VCV	3.6 ± 0.8	4.6 ± 1.1	4.6 ± 1.0	4.5 ± 1.1	0.265
	PCV	3.8 ± 0.7	4.5 ± 0.9	4.5 ± 0.8	4.6 ± 0.8	
Mean airway pressure, cmH_2_O	VCV	8 ± 1	11 ± 2 *	11 ± 1 *	9 ± 1	0.762
	PCV	9 ± 1	12 ± 1 *	12 ± 2 *	10 ± 1	
E_T_CO_2_, mmHg	VCV	33 ± 3	37 ± 2 *	37 ± 2 *	38 ± 3 *	0.552
	PCV	34 ± 3	37 ± 2 *	37 ± 2 *	38 ± 3 *	
Measured plateau pressure, cmH_2_O	VCV	11 ± 3	18 ± 3 *	18 ± 3 *	12 ± 2	
	PCV	NA	NA	NA	NA	
Dynamic compliance, mL/cmH_2_O	VCV	39.7 ± 10.8	21.7 ± 4.5 *	22.5 ± 6.1 *	35.2 ± 8.1	0.524
	PCV	45.6 ± 12.6	28.3 ± 7.0 *	30.7 ± 7.1 *	43.1 ± 10.5	

Values are presented as the mean ± SD. VCV, volume-controlled ventilation; PCV, pressure-controlled ventilation-volume guarantee; IND, 10 min after anesthesia induction; PP15 and PP30, 15 and 30 min after pneumoperitoneum induction; END, the end of surgery; E_T_CO_2_, end-tidal carbon dioxide tension; Group * time, aspect of parameter change over time; NA, not applicable. * *p* < 0.05, vs. IND within the group.

## Data Availability

Data are contained within the article.
